# Dentists with a physician or dentist parent: examining trends, challenges, and life satisfaction

**DOI:** 10.1186/s12909-025-07925-x

**Published:** 2025-10-15

**Authors:** Sydney L. Fu, Sean O. Fu, Rebecca Y. Chen, Enyi Jen, Min-Wen Fu, Hsun-Liang Chan, Earl Fu, Martin M. Fu

**Affiliations:** 1 Xiugang Campus, Kang Chiao International School, New Taipei City, Taiwan; 2 Department of Dentistry, Tri-Service General Hospital, National Defense Medical University, Taipei, Taiwan; 3 Department of Dentistry, Taipei Tzu Chi Hospital, New Taipei City, Taiwan; 4 Bridges Graduate School of Cognitive Diversity in Education, Studio City, CA USA; 5 Department of Endodontics, New York University College of Dentistry, New York, NY USA; 6 Division of Periodontology, College of Dentistry, The Ohio State University, Columbus, OH USA; 7 Department of Periodontics and Oral Medicine, School of Dentistry, University of Michigan, Ann Arbor, MI USA

**Keywords:** Psychological well-being, Happiness, Satisfaction, Career burnout, Career choice, Parent-child relations, Intergenerational relations

## Abstract

**Background:**

Dentists with a dentist parent are often assumed to have inherent career advantages. Despite the high prevalence of these “second-generation” dentists, little research has examined whether they are actually happier than others. This study investigated life satisfaction among dentists with a dentist parent, compared to those with a physician parent or non-physician/dentist parents.

**Methods:**

A cross-sectional survey was conducted among Taiwanese dentists, assessing their life satisfaction using the 5-item Satisfaction With Life Scale (SWLS), along with sociodemographic and career-related factors. Descriptive statistics and multiple linear regression analyses were performed to identify factors associated with SWLS scores.

**Results:**

Among 1,170 respondents (mean age = 43.5 years, SD = 12.0; 46.5% female), the prevalence of young dentists with at least one dentist parent increased from 2 to 10% over the past two decades, whereas the prevalence of dentists with a physician parent remained around 10%. Multivariable analysis revealed that dentists with a dentist parent reported lower mean SWLS scores (β = -0.245; *p* = 0.015) after accounting for potential confounders. When the items of the SWLS scale were analyzed individually, dentists with a dentist parent had similar current life satisfaction, but lower past life satisfaction (β = -0.541; *p* < 0.001) than those without physician/dentist parents. Among dentists who ranked in the top 25% of their class, those with a dentist parent reported the lowest mean SWLS scores. In contrast, dentists with a physician parent did not experience the same decline in life satisfaction observed among second-generation dentists.

**Conclusion:**

Although the small sample size of second-generation dentists limits the strength of inferences, these findings highlight the growing prevalence of second-generation dentists in Taiwan, their regret over past choices, and the potential challenges unique to this group. Understanding the pressures faced by second-generation dentists may inform strategies to enhance their professional fulfillment and overall well-being.

## Background

The psychological well-being of healthcare professionals is not only vital for their personal mental health but also plays a critical role in maintaining the quality of patient care and the stability of the healthcare system [1, 2]. Among younger generations of medical and dental students, work-life balance and overall well-being have emerged as key considerations when selecting a career or specialty [3–5]. Given these priorities, understanding the factors that influence the psychological well-being of healthcare practitioners carries significant implications for workforce development and health policy. One of the most widely recognized indicators of well-being is life satisfaction [6, 7]. Studies have shown that lower life satisfaction among physicians may be associated with decreased quality of care [8] and increased likelihood of early retirement [9], underscoring the importance of well-being in sustaining a healthy medical workforce.

A diverse healthcare workforce is crucial not only for enhancing patient communication but also for improving the quality of medical education and research [10–15]. However, a lack of diversity persists in both medicine [16] and dentistry, where a significant percentage of children of physicians and dentists choose to follow their parent’s career paths, causing social reproduction. Studies from Switzerland, Germany, and the United States indicate that between 15 and 81% of young dentists have at least one parent who is also a dentist [17–19]. Similarly, recent data from Sweden suggest a growing prevalence of physicians with a physician parent [20]. Despite this trend, the prevalence of physicians with a physician parent has remained relatively stable at approximately 13–20% in Western countries over the past few decades [20–27].

Many “second-generation” dentists are drawn to the profession due to early exposure through their dentist parents [28] or perceived advantages, such as better work-life balance compared to physicians, financial security, work independence, social prestige, and altruistic opportunities [29]. Moreover, dentists with a parent in the profession may benefit from intergenerational career transfers, receiving early mentorship, career-specific experience, or even inheriting their parents’ dental practices [18, 26, 30, 31]. As a result, second-generation dentists are often assumed to have an inherent advantage in their careers [16, 32, 33]. However, little research has examined whether these perceived advantages translate into greater life satisfaction or if the pressures of following in a parent’s footsteps impose unique psychological burdens.

Management research suggests that having a parent in the same profession can restrict successors’ autonomy, make the successors feel constrained, and amplify their fears of failing to meet their parents’ expectations [34, 35]. Whether similar dynamics affect second-generation dentists as they enter the workforce remains unclear. This study seeks to address this gap by evaluating the life satisfaction of dentists with a dentist or physician parent compared to their peers without a parent in dentistry or medicine.

## Methods

### Design and participants

The study was conducted in accordance with the Declaration of Helsinki. This cross-sectional study was approved by the Institutional Review Board at the University of Michigan (No. HUM00155739). Informed consent was obtained from all participants without a signature. The survey, conducted in Traditional Chinese, was administered through an online survey platform (SurveyMonkey) and distributed via email and closed social media groups exclusive to Taiwanese dentists. Additionally, dentists attending annual dental conferences were randomly invited to participate by completing the same online questionnaires in the exhibition hall. Data collection was conducted between December 2018 and August 2020. Dentists were excluded if they were undergoing clinical training (such as residency or fellowship), were not alumni of one of Taiwan’s seven dental schools, or were residing abroad.

### Measures

Life satisfaction was evaluated using the Satisfaction with Life Scale (SWLS) [36], a validated five-item instrument designed to assess an individual’s overall cognitive evaluation of life satisfaction based on their own criteria [6, 7]. The SWLS has been extensively employed across cultures and is recognized as a core indicator of subjective well-being. Its brevity makes it particularly suitable for reducing respondent burden and enhancing completion rates in multi-domain surveys. In our study, the SWLS demonstrated excellent internal reliability (Cronbach’s alpha = 0.92). The five items of SWLS includes: “In most ways my life is close to my ideal”, “The conditions of my life are excellent.“, “I am satisfied with my life.“, “So far I have gotten the important things I want in life.“, and “If I could live my life over, I would change almost nothing”. A Chinese version of SWLS, using a 5-point Likert scale (1 = strongly disagree, 5 = strongly agree), was employed [37]. The final SWLS score was calculated as the mean of the five item scores, with higher scores indicating greater life satisfaction.

The SWLS was the first set of questions presented in the survey. Subsequent sections collected non-dental-related information, including age, gender, marital status, and parent’s occupation (categorized as a dentist, physician, or neither), as well as perceived health, perceived familial interactions, and perceived friendships. To reduce survey fatigue and improve response rates, perceived health, familial interactions, and friendships were assessed using single-item Likert scales, categorized into five ordinal levels. The survey also gathered dental career-related information, including class rank in dental school, specialization, primary workplace, and weekly clinical hours with patients over the past year.

### Data analysis

Incomplete questionnaires were excluded from the analysis. Bivariate analyses were conducted using either the Kruskal-Wallis test or the Mann-Whitney U test to identify associations between individual independent variables and SWLS scores. The relationship between dentists’ age and the prevalence of having a dentist or physician parent was examined with logistic regression. Multivariable analysis was performed via multiple linear regression to identify independent association with life satisfaction. All statistical analyses were conducted using SPSS Statistics (IBM Corp., NY, USA), and a significance level set at *p* < 0.05.

## Results

### Descriptive data

Email invitations were sent to 1,328 dentists, of whom 437 responded, resulting in a 32.9% email response rate. An additional 1,345 dentists participated through social media or at dental conferences, though the response rate for this group is undetermined. From the initial sample of 1,782 individuals, 396 were excluded for residing overseas, lacking a Taiwanese dental degree, or currently being in postgraduate training. Additionally, 216 respondents were excluded due to incomplete survey responses. Ultimately, 1,170 valid responses were included in the analysis. Considering that approximately 15,000 dentists in Taiwan are not engaged in active postgraduate training, the 1,170 participants of this convenience sample represent 7.8% of the total dentist population in the country.

Demographic characteristics, health, and social relationships are summarized in Table 1. All participants were divided into three different parental occupation groups: non-physician/dentist parents (*n* = 1,034), dentist parent (i.e., second-generation dentist) (*n* = 40), and physician parent (*n* = 96). Four participants who reported having both a physician and a dentist parent were assigned to the dentist parent group.

**Table 1:** Demographic, health, social, and career characteristics by parental occupations (*n* = 1,170). Values are presented as mean ± sd or number (%)

Parental occupations	≤	Non-physician/dentist (*n* = 1034)	*p*-value (Non-physician /dentist vs. Dentist)	Dentist (*n* = 40)	*p*-value (Physician vs. Dentist)	Physician (*n* = 96)
Age (in years)		44.0 ± 11.9	< 0.001	36.1 ± 10.6	0.006	41.6 ± 12.1
Age	20–29	89 (8.6)	< 0.001	11 (27.5)	0.008	12 (12.5)
	30–39	367 (35.5)		20 (50.0)		43 (44.8)
	40–49	234 (22.6)		4 (10.0)		16 (16.7)
	50–59	209 (20.2)		3 (7.5)		12 (12.5)
	60–69	135 (13.1)		2 (5.0)		13 (13.5)
Gender	Male	571 (44.8)	0.005	13 (32.5)	0.223	42 (43.8)
	Female	463 (55.2)		27 (67.5)		54 (56.3)
Marital status	Single/separated	219 (21.2)	0.005	16 (40.0)	0.106	25 (26.0)
	Married/cohabitated	815 (78.8)		24 (60.0)		71 (74.0)
Self-rated health	Very poor	5 (0.5)	0.019	0 (0.0)	0.128	2 (2.1)
	Poor	96 (9.3)		10 (25.0)		9 (9.4)
	Fair	411 (39.7)		15 (37.5)		40 (41.7)
	Good	458 (44.3)		12 (30.0)		40 (41.7)
	Very good	65 (6.2)		3 (7.5)		5 (5.2)
Interaction with family	Very poor	6 (0.6)	< 0.001	2 (5.0)	0.098	0 (0.0)
	Poor	23 (2.2)		4 (10.0)		6 (6.3)
	Fair	229 (22.1)		11 (27.5)		18 (18.8)
	Good	568 (54.9)		16 (40.0)		54 (56.3)
	Very good	208 (20.1)		7 (17.5)		18 (18.8)
Relationship with friends	Very poor	0 (0.0)	0.861	0 (0.0)	0.761	0 (0.0)
	Poor	11 (22.7)		0 (0.0)		1 (1.0)
	Fair	235 (9.7)		8 (20.0)		25 (2.0)
	Good	646 (62.5)		27 (67.5)		57 (59.4)
	Very good	142 (13.7)		5 (12.5)		13 (13.5)
Class rank in dental school	Top 25%	465 (45.0)	0.782	18 (45.0)	0.650	37 (38.5)
	26–50%	306 (29.6)		10 (25.0)		31 (32.3)
	Bottom 50%	263 (25.5)		12 (30.0)		28 (29.2)
Specialty	Specialist	553 (53.5)	0.002	12 (30.0)	< 0.001	63 (65.6)
	General dentist	481 (46.5)		28 (70.0)		33 (34.4)
Main employment status	Private (co-)owner	497 (48.6)	0.925	11 (27.5)	0.567	42 (43.7)
	Private employee	357 (34.5)		22 (55.0)		33 (34.4)
	Hospital or school	180 (17.4)		7 (17.5)		21 (21.9)
Clinical hours per week	< 20 h	128 (12.4)	0.072	9 (22.5)	0.688	19 (19.8)
	20–29 h	210 (20.3)		9 (22.5)		18 (18.8)
	30–39 h	371 (35.9)		13 (32.5)		36 (37.5)
	≥ 40 h	325 (31.4)		9 (22.5)		23 (24.0)
Satisfaction With Life Scale		3.63 ± 0.76	< 0.001	3.13 ± 0.65	0.001	3.58 ± 0.81

**Fig. 1:**
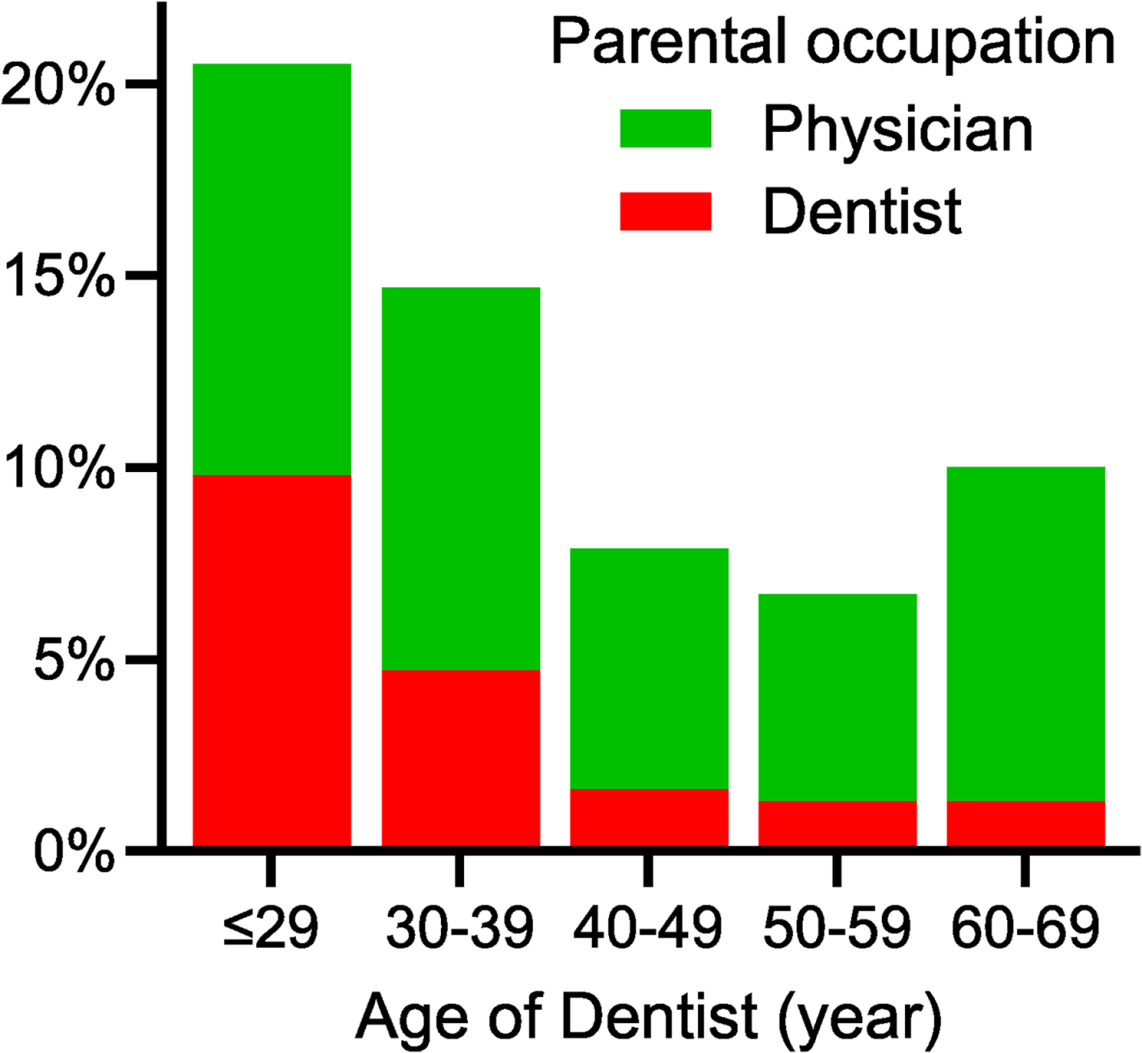
Prevalence of Taiwanese dentists with at least one dentist or physician parent by age groups

Participants with a dentist parent were significantly younger (mean age = 36.1 ± 10.6 years) compared to those in the other two groups (*p* < 0.001 and *p* = 0.006) (Table 1). Among participants under 30 years of age, 9.8% had a dentist parent, while 10.7% had a physician parent (Fig. 1). The percentage of second-generation dentists decreased significantly with age (*p* < 0.001). In contrast, the percentage of dentists with a physician parent remained relatively stable across different ages (*p* = 0.064). Participants with a dentist parent also reported poor self-rated health (*p* = 0.019) and less favorable familial interactions (*p* < 0.001) compared to those with non-physician/dentist parents (Table 1).

### Bivariate analysis of life satisfaction

**Fig. 2:**
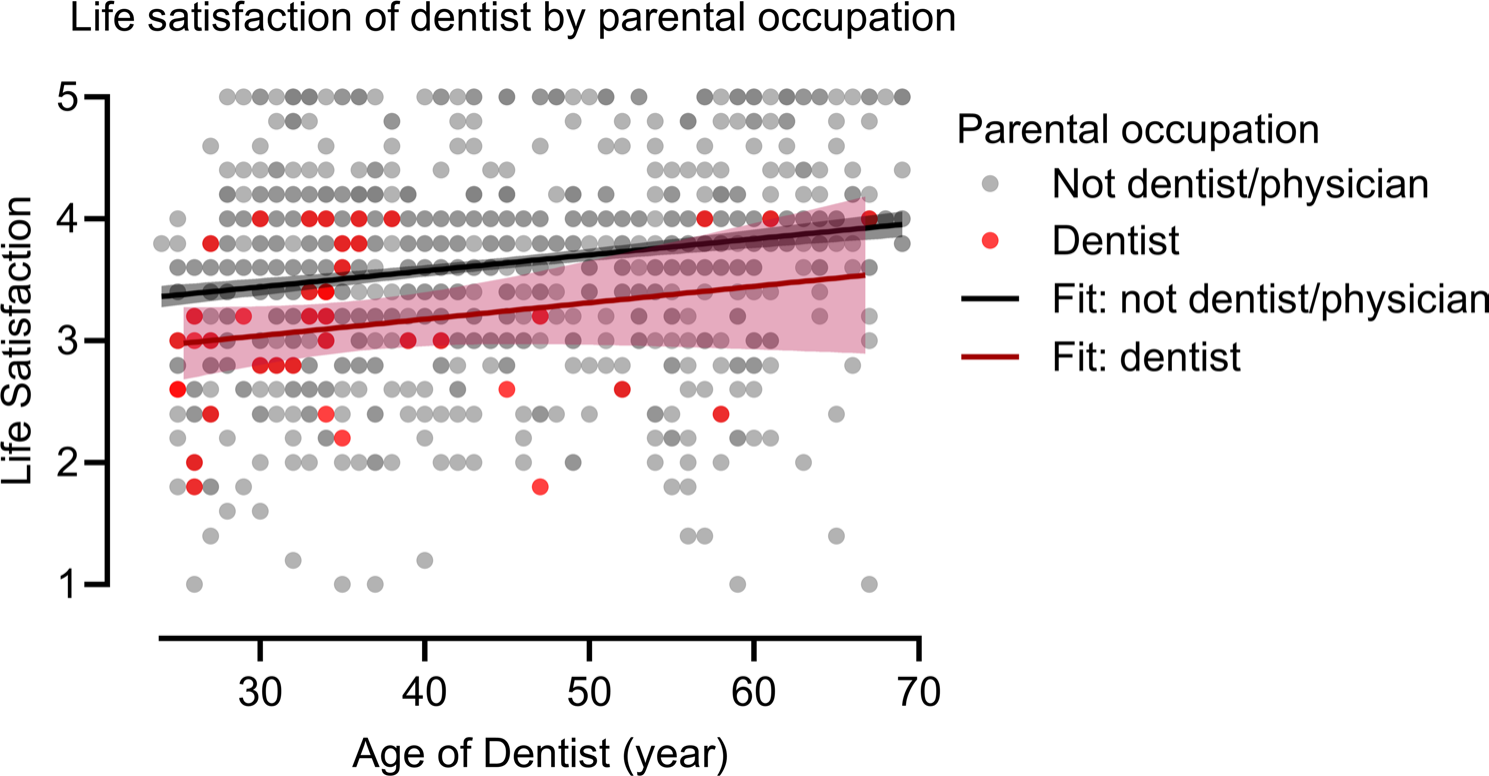
SWLS scores by dentist parent status: scatter plot with linear regression lines and 95% confidence intervals. Compares life satisfaction among dentists with (red; *n* = 40) and without a dentist parent (black; *n* = 1034)

**Fig. 3:**
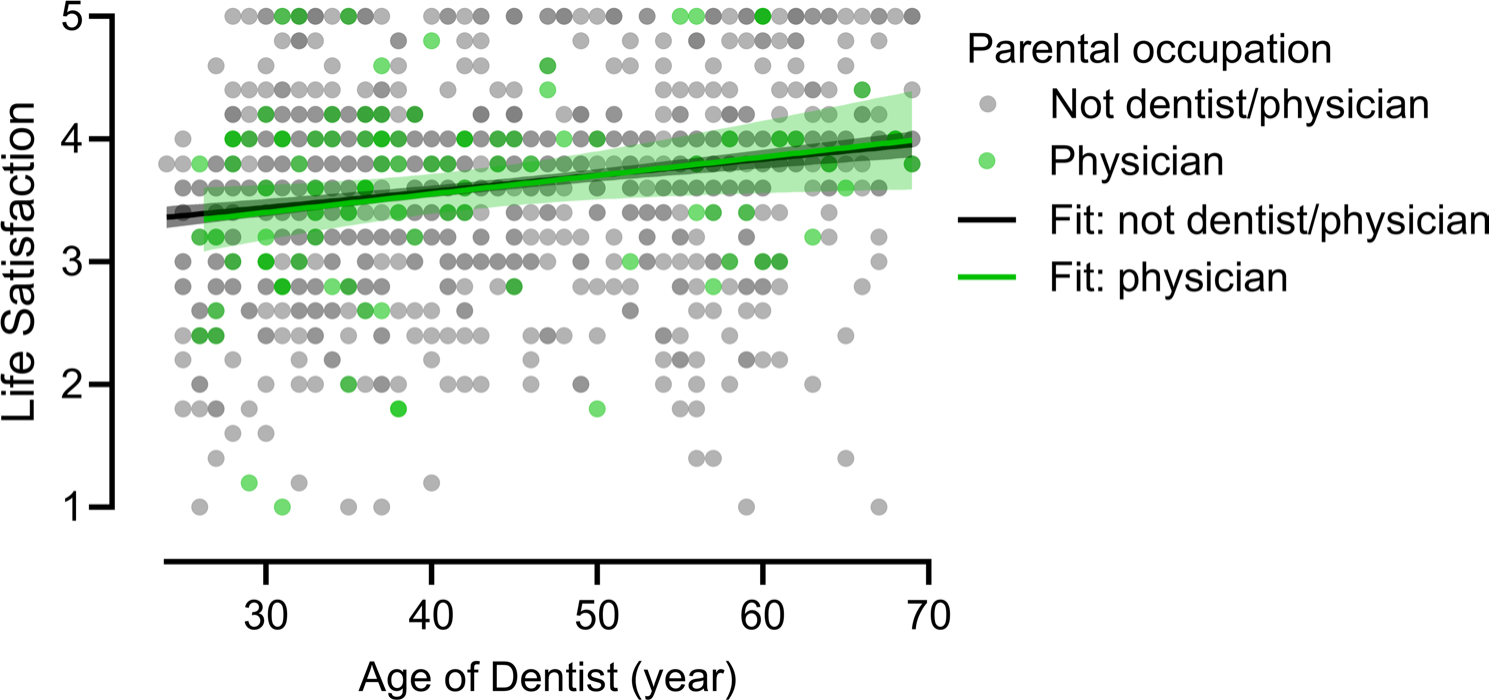
SWLS scores by physician parent status: scatter plot with linear regression lines and 95% confidence intervals. Compares life satisfaction among dentists with (green; *n* = 96) and without a physician parent (black; *n* = 1034)

Participants with a dentist parent reported lower mean SWLS scores (3.13 ± 0.65) compared to those with non-physician/dentist parents (3.63 ± 0.76, *p* < 0.001) and those with a physician parent (3.58 ± 0.81, *p* = 0.001) (Table 1). The mean SWLS scores by parental occupations and examined characteristics are presented in Table 2. Scatter plots with linear regression fits illustrated the distribution (Figs. 2 and 3). Notably, no participants with a dentist parent had a SWLS score above 4.0, whereas 12% of participants in the other two groups did.

**Table 2:** Mean satisfaction with life scale (SWLS) scores across different sociodemographic and career-related variables by parental occupation

Parental occupations		Non- physician/dentist (*n* = 1034)		Non- physician/ dentist vs. Dentist	Dentist (*n* = 40)		Physician vs. Dentist	Physician (*n* = 96)	
		Mean	SD	*p*-value	Mean	SD	*p*-value	Mean	SD
Age, y	20–29	3.19	0.81	0.071	2.78	0.58	0.190	3.13	0.87
	30–39	3.58	0.73	0.090	3.34	0.56	0.275	3.49	0.87
	≥ 40	3.72	0.74	0.027	3.07	0.80	0.028	3.80	0.68
Gender	Male	3.62	0.78	0.040	3.15	0.76	0.137	3.54	0.98
	Female	3.63	0.73	< 0.001	3.11	0.61	0.002	3.61	0.66
Marital status	Single/separated	3.35	0.70	0.033	2.96	0.62	0.404	3.13	0.81
	Married/cohabitated	3.70	0.75	0.002	3.23	0.66	0.004	3.74	0.76
Self-rated health	(Very) poor	2.98	0.78	0.063	2.54	0.58	0.387	2.95	0.87
	Fair	3.39	0.70	0.004	2.95	0.45	0.007	3.49	0.89
	(Very) good	3.93	0.64	0.082	3.69	0.38	0.507	3.81	0.63
Interaction with family	(Very) poor	2.64	0.78	0.480	2.43	0.46	0.394	2.87	1.06
	Fair	3.15	0.69	0.348	2.95	0.63	0.204	3.27	0.86
	Very good	3.80	0.68	0.003	3.39	0.55	0.047	3.71	0.74
Relationship with friends	(Very) poor	2.75	0.54	--	--	--	--	1.20	--
	Fair	3.26	0.73	0.057	2.80	0.60	0.017	3.47	0.63
	(Very) good	3.75	0.72	< 0.001	3.21	0.65	0.003	3.65	0.83
Class rank in dental school	Top 25%	3.67	0.73	< 0.001	2.83	0.59	< 0.001	3.81	0.64
	26–50%	3.66	0.72	0.657	3.48	0.71	0.653	3.71	0.68
	Bottom 50%	3.50	0.83	0.175	3.27	0.54	0.942	3.11	0.97
Specialization	Specialist	3.73	0.70	0.033	3.23	0.75	0.049	3.74	0.79
	General dentist	3.51	0.80	0.002	3.08	0.62	0.185	3.27	0.78
Main employment	Private (co-)owner	3.70	0.74	0.044	3.16	0.79	0.059	3.72	0.92
	Private employee	3.59	0.77	0.009	3.17	0.67	0.324	3.35	0.77
	Hospital or school	3.49	0.76	0.010	2.91	0.30	0.004	3.65	0.59
Clinical hours per week	< 20 h	3.85	0.75	0.111	3.42	0.67	0.188	3.87	0.68
	20–29 h	3.81	0.67	0.042	3.33	0.52	0.403	3.61	0.67
	30–39 h	3.58	0.73	0.002	3.00	0.59	0.013	3.53	0.70
	≥ 40 h	3.40	0.79	0.014	2.80	0.74	0.086	3.38	1.11

When analyzing class rank in dental school (Table 2; Fig. S1), participants ranked in the top 25% of their class with a dentist parent reported the lowest life satisfaction (mean = 2.83, SD = 0.59). Within the group with a dentist parent, those ranked in the top 25% had significantly lower SWLS scores than the 26–50% group (*p* = 0.031).

### Multivariable analysis of life satisfaction

Multiple linear regression analysis indicated that dentists with a dentist parent had lower mean SWLS scores than those with the non-dentist/physician group (*β* = 0.245, 95% CI [0.048–0.442], *p* = 0.015) and those with a physician parent (*β* = 0.234, 95% CI [0.05–0.463], *p* = 0.045) after accounting for the examined factors (Table 3).

**Table 3:** Multiple linear regression analysis of overall SWLS mean scores by parental occupation and covariates (*n* = 1,170)

	Life Satisfaction	
Constant	0.33	--
Parents’ occupations		
Not dentist nor physician	0.25 (0.05 to 0.44)	0.015
Medical doctor	0.23 (0.01 to 0.46)	0.045
Dentist	Reference	
Age	0.01 (0.00 to 0.01)	< 0.001
Gender		
Female	0.06 (−0.03 to 0.15)	0.162
Male	Reference	
Marital status		
Married, cohabited	0.16 (0.07 to 0.26)	0.001
Single, separated, divorced	Reference	
Self-rated health^a^	0.26 (0.21 to 0.31)	< 0.001
Interaction with family^a^	0.26 (0.20 to 0.31)	< 0.001
Relationship with friends^a^	0.09 (0.03 to 0.16)	0.005
Class rank in dental school		
Top 25%	0.05 (−0.04 to 0.14)	0.317
26–50%	0.06 (−0.03 to 0.16)	0.207
Bottom 50%	Reference	
Specialization qualification		
Specialist	0.11 (0.04 to 0.19)	0.004
General dentist	Reference	
Main employment status		
Private practice (co-)owner	0.13 (0.02 to 0.24)	0.018
Private practice employee	0.12 (0.01 to 0.22)	0.032
Hospital or school	Reference	
Clinical hours per week		
< 20 h	0.14 (0.01 to 0.27)	0.031
20–29 h	0.15 (0.04 to 0.26)	0.006
30–39 h	0.10 (0.01 to 0.19)	0.023
≥ 40 h	Reference	

^a^Scores range from 1 to 5, with higher scores indicating better health, interaction, or relationship

When the five items of the SWLS scale were analyzed individually (Table 4), dentists with a dentist parent scored lower on item 5 (“If I could live my life over, I would change almost nothing”) compared to those with non-dentist/physician parents (β = 0.541, 95% CI [0.282 to 0.802], *p* < 0.001) and those with a physician parent (β = 0.526, 95% CI [0.230 to 0.834], *p* = 0.001) after accounting for examined factors. Dentists with a dentist parent also scored lower on Item 3 (“So far I have gotten the important things I want in life”) compared to those with non-dentist/physician parents (β = 0.251, 95% CI [0.001 to 0.502], *p* = 0.049).

**Table 4:** Multiple linear regression analysis of individual SWLS items by parental occupation (*n* = 1,170)*

	Item 1: In most ways my life is close to my ideal.		Item 2: The conditions of my life are excellent.		Item 3: So far I have gotten the important things I want in life.		Item 4: I am satisfied with my life.		Item 5: If I could live my life over, I would change almost nothing.	
	β (95%CI)	*p*	β (95%CI)	*p*	β (95%CI)	*p*	β (95%CI)	*p*	β (95%CI)	*p*
Constant	− 0.067	--	0.612	--	0.018	--	0.541	--	0.562	--
Parental occupation										
Not dentist/physician	0.17 (−0.08 to 0.42)	0.174	0.12 (−0.09 to 0.34)	0.268	0.25 (0.00 to 0.50)	0.049	0.14 (−0.08 to 0.36)	0.215	0.54 (0.28 to 0.80)	< 0.001
Physician	0.26 (0.02 to 0.55)	0.071	0.09 (−0.16 to 0.34)	0.482	0.17 (−0.12 to 0.46)	0.261	0.15 (−0.10 to 0.42)	0.244	0.53 (0.23 to 0.83)	0.001
Dentist	Reference		Reference		Reference		Reference		Reference	

*adjusted with age, gender, health, family interaction, friends relationship, class rank in dental school, specialization, employment status, and clinical hours per week

For the dentists in the top 25% of their class in dental school (Table S1), those with a dentist parent had a marginal significantly lower mean SWLS score compared to dentists with non-dentist/physician parents (β = 0.272, 95% CI [−0.021 to 0.565], *p* = 0.068) and a significantly lower mean SWLS score compared to those with a physician parent (β = 0.560, 95% CI [0.212 to 0.908], *p* = 0.002).

## Discussion

This study examined the life satisfaction of dentists in Taiwan with at least a dentist or physician parent. Through multivariable analysis, we found that: (a) second-generation dentists expressed lower satisfaction with their past life than those without a dentist parent; and (b) dentists with a physician parent did not experience the same decline in life satisfaction observed among second-generation dentists. These findings underscore the need to explore the psychological impact of intergenerational career pathways in dentistry and to identify potential strategies to mitigate the challenges faced by second-generation dentists.

### Trends in second-generation dentists across countries

Studies have reported varying prevalences of dental students with a dentist parent across the globe. Data from over 20 years ago indicated a prevalence rate of 7% in Iran [38] and 20% in the United States [18]. More recent national surveys reported that 15% of German dental students [19] and 81% of Swiss young dentists have at least one parent who is also a dentist [17]. Similarly, our study identified an increasing prevalence of dentists with a dentist parent in Taiwan, rising from 1.3% among dentists aged 50–69 to 9.8% among those under 30 (Fig. 1). Our findings provide further evidence of social reproduction, which limits the entry of underrepresented groups into the profession and diminishes diversity within the field.

### Psychological well-being of dental students with a dentist parent

Although dentists with a dentist parent have become increasingly common, research on the psychological well-being of this group remains limited. Most studies have primarily focused on dental students. A recent qualitative interview study highlighted that having a dentist parent is perceived as an advantage due to career-specific intergenerational transfers [29]. However, a recent study revealed that young dentists in Switzerland with a dentist parent viewed their career prospects as marginal significantly less optimistic than their counterparts without a dentist parent [17]. Their finding aligns with our observation of reduced past life satisfaction among second-generation dentists in the present study. Similarly, research on the career choice motives of Iranian dental students found that, compared with their classmates without a dentist parent, senior dental students with a dentist parent were more aware of the profession’s challenges before applying to dental school. However, they were less interested in hands-on work, less enthusiastic about dentistry, and more likely to have chosen dentistry due to strong parental recommendation [38].

### Psychological well-being of medical students with a physician parent

Similar trends have been observed among medical students. A study conducted at a public university in Japan found that first-year medical students with a parent who is medical professional exhibited lower academic motivation compared to those from non-medical families [39]. Additionally, studies from South Asia and the Middle East also revealed that medical students with a physician parent faced more academic pressure [40], higher rates of burnout [41], and poorer sleep quality [42] compared to their classmates without a physician parent. These studies suggest that elevated parental expectations or intensified pressure to meet their parents’ standards may contribute to their psychological challenges, especially when students feel they are falling short.

However, parental expectations alone cannot explain why dentists with a physician parent did not experience a similar decline in past life satisfaction, as both physician and dentist parents likely hold comparable expectations for their children. Furthermore, previous studies on medical students have not examined whether the impact of parental expectations persists to the same extent after graduation and entry into the workforce.

### Autonomy and decision-making constraints

Outside the healthcare field, a study comparing founders and successors in family business found that successors may report lower entrepreneurial job satisfaction than founders [35]. This discrepancy in job satisfaction was attributed to successors’ perceived lower degree of strategic decision-making freedom compared to founders [43]. Autonomy, flexibility, and independence at work are well-known factors closely associated with job satisfaction [44, 45]. Constraints on decision-making freedom and limited autonomy imposed by dentist parents may explain the reduced past life satisfaction observed in second-generation dentists, in contrast to the unchanged life satisfaction of those with a physician parent. The distinct nature of the physician and dentist professions may prevent physician parents from restricting their dentist children’s autonomy and strategic decision-making freedom.

Similarly, studies have shown that the careers of successors in family business may also be burdened or constrained by familial and social relationships [35, 46]. Successors may prioritize maintaining social status or preserving wealth over pursuing other, potentially riskier career opportunities they might have originally been interested in [35, 43, 47]. Founders may also influence successors by imposing their definition of success or unintentionally demonstrating mistrust in the successor’s professional abilities [48]. As a result, successors are likely to remain in the shadow of the founder, driven by constant comparisons to the predecessor [35, 49].

### High expectation and social comparisons in career satisfaction

People often evaluate their overall circumstances in relation to a reference group, such as their parents [50, 51]. In Taiwan, dentists typically have strong academic qualifications or scores in high school that allow them to gain admission to almost any college major, except for a few top-tier medical schools. Consequently, some first-generation dentists who chose not to follow their parents’ career paths might feel fortunate or grateful that they do not have to replicate their parents’ lives. In contrast, dentists with a dentist parent may initially envision achieving a successful career and fulfilling lives similar to their parents. However, after replicating their parent’s past strategies, they might either fail to attain the same level of success or not experience a significant improvement in life after entering the workforce [34]. In other words, second-generation dentists may hold higher expectations for their lives due to a higher reference group compared to first-generation dentists. This phenomenon is supported by a panel study, which found that second-generation self-employed individuals in the UK and France reported lower job satisfaction than their first-generation self-employed counterparts [34].

### Dissatisfaction with past life

When the five items of SWLS were examined individually, we found that dentists with a dentist parent scored significantly lower on the third item of SWLS (“So far I have gotten the important things I want in life”) and the fifth item (“If I could live my life over, I would change almost nothing”) compared to dentists without physician or dentist parents in multivariate analysis (Table 4). Unlike the other three items, which focus on external living conditions or current levels of satisfaction, the third and fifth items assess satisfaction with past accomplishments or regret rather than present circumstances [6, 7, 52]. These findings suggest that, while dentists with a dentist parent are satisfied with their present lives, they have more regret over past choices and a stronger desire to make changes if given the opportunity to relive their lives. Unfortunately, since the SWLS relies on respondents to consciously evaluate and judge their overall life satisfaction based on their own criteria or standards [6, 7], the present study lacked sufficient information to identify which specific changes dentists with a dentist parent would make if they could relive their lives.

### Top 25% of the class in dental school

In addition, we observed that the dentists ranked in the top 25% in their dental class who have a dentist parent scored the lowest on SWLS (Fig. S1 and Table S1). These findings suggest that academic performance may influence life satisfaction differently across parental occupation groups. A study of Mensa members, individuals with IQ scores in the 98th percentile or higher, found that gifted adults were happiest with their work and life when they owned their own businesses or held leadership roles that offered substantial autonomy [53]. Autonomy has also been identified as a key factor influencing job satisfaction for physicians [54] and for successors in family business as mentioned earlier. Moreover, research shows that gifted adolescents with multiple talents often fear making the wrong career choice or “wasting their talent”. Consequently, they may yield to parental or societal pressure when choosing a career, prioritizing income, respect, and prestige over personal interest and satisfaction [55]. Choosing a career that does not align with their original interests, coupled with insufficient autonomy, might explain the low life satisfaction of second-generation dentists who ranked in the top 25% of their dental class.

### Dentists with a physician parent

One more notable finding from the present study is that dentists with a physician parent did not have the same decline in life satisfaction observed among dentists with a dentist parent. In upper-middle-income countries, it has been identified that financial security, respected social status, and parental expectation are the primary reasons medical students choose to study medicine, rather than consideration of work-life balance [56]. In response to the high prevalence of burnout and career regret among physicians [57], some physician parents may later encourage their children to prioritize lifestyle and family when selecting careers [58]. Dentists are generally known to experience lower levels of burnout and better work-life balance compared to physicians [59–64] while still enjoying compatible social prestige, financial security, and work independence. Dentists with a physician parent may feel that they have fulfilled their parents’ expectations for a respectable and successful career while avoiding the levels of burnout their parents endured. This perception could contribute to their undeclined life satisfaction. This unique dynamic may partly explain why dentists with a physician parent report greater life satisfaction than those with a dentist parent.

### Limitations

This study has several limitations. First, the sample size of the group with a dentist parent aged above 40 years old is significantly smaller compared to the other two groups, which may affect the reliability of the analysis and interpretation. However, this age discrepancy reflects the relative rarity of middle-aged dentists in Taiwan who have a parent sharing the same profession, as evidenced by the prevalence of dentists in Taiwan being 0.02% of the general population in 1986, but increased 3.6-fold to 0.07% in 2023 [65]. Additionally, studies investigating the psychological well-being of middle-aged dentists remain globally rare. The inclusion of this underdeveloped group allowed for a more comprehensive understanding of the issue. Second, cross-cultural psychology research has shown that East Asians tend to evaluate their personal accomplishments or task performance less positively than Americans [52]. Asians are also more likely to base their career choices on culture, familiarity, and community rather than personal preferences — a trend less common in Western nations [66–68]. Since this study included only Taiwanese participants, its findings may not be generalizable to dentists in other nations. Third, the cross-sectional design of the study limits its ability to establish causal relationships. Longitudinal studies are necessary to confirm our findings and clarify the trajectory of life satisfaction. Fourth, the use of convenient sampling may limit the representativeness of the study population, potentially affecting the generalizability of the results. Fifth, from a practical career-choice perspective, it remains unclear whether individuals with a dentist parent would have been happier had they pursued a different career path. Sixth, the survey did not collect detailed information on the relationship between second-generation dentists and their dentist parents, such as whether they pursued the same specialty, began their careers in their parents’ dental practices, or had one or both parents who were dentists. These familial configurations may have distinct psychological associations with autonomy and life satisfaction.

## Conclusion

Due to the small sample size of second-generation dentists, only limited conclusions can be drawn. However, this study highlights that dentists with a dentist parent, particularly those with strong academic performance, reported lower satisfaction with their past life compared to their peers without a dentist parent. In contrast, dentists with a physician parent did not exhibit the same decline in past life satisfaction. With the increasing presence of second-generation dentists, their issue of more regret over past choices warrants greater attention, as addressing healthcare providers’ challenges may contribute to both the stability of the healthcare workforce and the quality of care.

## Supplementary Information

Supplementary Fig. 1. The*Satisfaction With Life Scale* mean scores by different class rank and parental occupations.

Supplementary Table 1. Multiple linear regression analysis of overall SWLS mean scores by parental occupation and class rank(n= 1,170)*.

## Data Availability

All data used and/or analyzed during this study are available from the corresponding author upon reasonable request.

## References

[1] Tawfik DS, Scheid A, Profit J, Shanafelt T, Trockel M, Adair KC, et al. Evidence relating health care provider burnout and quality of care: a systematic review and meta-analysis. Ann Intern Med. 2019;171(8):555–67. 31590181 10.7326/M19-1152 PMC7138707

[2] Hodkinson A, Zhou A, Johnson J, Geraghty K, Riley R, Zhou A, Panagopoulou E, Chew-Graham CA, Peters D, Esmail A, et al. Associations of physician burnout with career engagement and quality of patient care: systematic review and meta-analysis. Br Med J. 2022;378:e070442. 36104064 10.1136/bmj-2022-070442 PMC9472104

[3] Kaiser KA, Lench HC, Levine LJ. Medical residency match applicants undervalue factors that predict stress and burnout. Med Educ Online. 2022;27(1):2109243. 35946069 10.1080/10872981.2022.2109243 PMC9373742

[4] Yang Y, Li J, Wu X, Wang J, Li W, Zhu Y, et al. Factors influencing subspecialty choice among medical students: a systematic review and meta-analysis. BMJ Open. 2019;9(3):e022097. 10.1136/bmjopen-2018-022097 PMC6429728 30850399

[5] Harrison JL, Platia CL, Ferreira L, Soh M, Bugueno JM, Thompson TL, et al. Factors affecting dental students’ postgraduate plans: a multi-site study. J Dent Educ. 2022;86(2):124–35. 34554565 10.1002/jdd.12792

[6] Pavot W, Diener E. The satisfaction with life scale and the emerging construct of life satisfaction. J Posit Psychol. 2008;3(2):137–52.

[7] Pavot W, Diener E. Review of the satisfaction with life scale. Psychol Assess. 1993;5(5):164–72.

[8] Leigh JP, Tancredi DJ, Kravitz RL. Physician career satisfaction within specialties. BMC Health Serv Res. 2009;9:166. 19758454 10.1186/1472-6963-9-166 PMC2754441

[9] Pedrazza M, Berlanda S, Trifiletti E, Bressan F. Exploring physicians’ dissatisfaction and work-related stress: development of the phydis scale. Front Psychol. 2016;7:1238. 27588013 10.3389/fpsyg.2016.01238 PMC4988987

[10] Clayborne EP, Martin DR, Goett RR, Chandrasekaran EB, McGreevy J. Diversity pipelines: the rationale to recruit and support minority physicians. JACEP Open. 2021;2(1):e12343. 33532751 10.1002/emp2.12343 PMC7823093

[11] Marrast LM, Zallman L, Woolhandler S, Bor DH, McCormick D. Minority physicians’ role in the care of underserved patients: diversifying the physician workforce may be key in addressing health disparities. JAMA Intern Med. 2014;174(2):289–91. 24378807 10.1001/jamainternmed.2013.12756

[12] Stanford FC. The importance of diversity and inclusion in the healthcare workforce. J Natl Med Assoc. 2020;112(3):247–9. 32336480 10.1016/j.jnma.2020.03.014 PMC7387183

[13] Cooper LA, Roter DL, Johnson RL, Ford DE, Steinwachs DM, Powe NR. Patient-centered communication, ratings of care, and concordance of patient and physician race. Ann Intern Med. 2003;139(11):907–15. 14644893 10.7326/0003-4819-139-11-200312020-00009

[14] Snyder JE, Upton RD, Hassett TC, Lee H, Nouri Z, Dill M. Black representation in the primary care physician workforce and its association with population life expectancy and mortality rates in the US. JAMA Netw Open. 2023;6(4):e236687. 37058307 10.1001/jamanetworkopen.2023.6687 PMC10105312

[15] Silver JK, Bean AC, Slocum C, Poorman JA, Tenforde A, Blauwet CA, et al. Physician workforce disparities and patient care: a narrative review. Health Equity. 2019;3(1):360–77. 31312783 10.1089/heq.2019.0040 PMC6626972

[16] Wouters A, Croiset G, Isik U, Kusurkar RA. Motivation of Dutch high school students from various backgrounds for applying to study medicine: a qualitative study. BMJ Open. 2017;7(5):e014779. 10.1136/bmjopen-2016-014779 PMC5623448 28576893

[17] Campus G, Rusca P, Amrhein C, Meier A, Zeyer O, Wolf TG. Career prospects of young dentists in Switzerland. Int J Environ Res Public Health. 2020. 10.3390/ijerph17124310. 10.3390/ijerph17124310 PMC7345911 32560199

[18] Park SE, Da Silva JD, Barnes JL, Susarla SM, Howell TH. Predicting dental school performance based on prior dental experience and exposure. Eur J Dent Educ. 2010;14(1):1–6. 10.1111/j.1600-0579.2009.00611.x 20070792

[19] Thiem DGE, Puladi B, Seifert L, Becker P, Bjelopavlovic M, Magennis P, Wiltfang J, Warwas FB. Post-graduation career pathways: a nationwide survey among dental students in Germany. Clin Oral Investig. 2024;28(2):134. 10.1007/s00784-024-05535-3 PMC10844428 38316644

[20] Polyakova M, Persson P, Hofmann K, Jena AB. Does medicine run in the family-evidence from three generations of physicians in sweden: retrospective observational study. Br Med J. 2020;371:m4453. 33328192 10.1136/bmj.m4453 PMC7737652

[21] Xu G, Veloski JJ. Physician parents’ influence over their children’s choices of careers in generalist specialties. Acad Med. 1998;73(8):913. 10.1097/00001888-199808000-00020 9736855

[22] O’Neill L, Vonsild MC, Wallstedt B, Dornan T. Admission criteria and diversity in medical school. Med Educ. 2013;47(6):557–61. 23662872 10.1111/medu.12140

[23] Heath CC, Stoddart C, Green H. Parental backgrounds of Otago medical students. N Z Med J. 2002;115(1165):U237. 12552284

[24] Gude T, Vaglum P, Tyssen R, Ekeberg O, Hem E, Rovik JO, et al. Identification with the role of doctor at the end of medical school: a nationwide longitudinal study. Med Educ. 2005;39(1):66–74. 15612902 10.1111/j.1365-2929.2004.02034.x

[25] Gude T, Vaglum P. Parent being a physician: any influence upon job stress in young physicians? A Norwegian prospective and longitudinal cohort study. Patient Educ Couns. 2017;100(11):2144–6. 28647063 10.1016/j.pec.2017.06.018

[26] Finset KB, Gude T, Hem E, Tyssen R, Ekeberg O, Vaglum P. Which young physicians are satisfied with their work? A prospective nationwide study in Norway. BMC Med Educ. 2005;5(1):19. 15932648 10.1186/1472-6920-5-19 PMC1164416

[27] Schwartz MD, Linzer M, Babbott D, Divine GW, Broadhead E. Medical student interest in internal medicine. Initial report of the society of general internal medicine interest group survey on factors influencing career choice in internal medicine. Ann Intern Med. 1991;114(1):6–15. 1983935 10.7326/0003-4819-114-1-6

[28] Yang C, Jin X, Yan J, Zhang J, Chen C, Cheng Y, et al. An investigation of the intention and reasons of senior high school students in China to choose medical school. BMC Med Educ. 2021;21(1):242. 33902559 10.1186/s12909-021-02677-w PMC8077942

[29] Cheng FC, Wang LH, Wang YC, Chiang CP. The influence of dentist parents on their children’s career decision-making for dentistry or medicine. J Dent Sci. 2024;19(1):678–81. 38303809 10.1016/j.jds.2023.10.003 PMC10829773

[30] Khair D, Blanchard CC, Wang KK, Moore DB. The matthew effect: prevalence of doctor and physician parents among ophthalmology applicants. J Acad Ophthalmol. 2023;15(2):e295-9. 10.1055/s-0043-1777432 PMC10723966 38107879

[31] Gough HG, Hall WB. A comparison of medical students from medical and nonmedical families. J Med Educ. 1977;52(7):541–7. 874986 10.1097/00001888-197707000-00001

[32] Wright SR, Boyd VA, Okafor I, Sharma M, Giroux R, Richardson L, et al. First in family’ experiences in a Canadian medical school: a critically reflexive study. Med Educ. 2023;57(10):980–90. 37226410 10.1111/medu.15116

[33] Aboudeif A, Elaraby Y, Hany M, Nasser S, Refaat N, Mohamed YG, et al. Inherited privilege? First vs. continuing-generation medical students in Egypt, academic performance, extracurricular training and expectations: a cross-sectional study. BMC Med Educ. 2024;24(1):1274. 39506796 10.1186/s12909-024-06227-y PMC11542418

[34] Clark A, Colombier N, Masclet D. Never the same after the first time: the satisfaction of the second-generation self‐employed. Int J Manpow. 2008;29(7):591–609.

[35] Lauto G, Pittino D, Visintin F. Satisfaction of entrepreneurs: a comparison between founders and family business successors. J Small Bus Manage. 2020;58(3):474–510.

[36] Diener E, Emmons RA, Larsen RJ, Griffin S. The satisfaction with life scale. J Pers Assess. 1985;49(1):71–5. 16367493 10.1207/s15327752jpa4901_13

[37] Yeung GTY, Fung HH. Social support and life satisfaction among Hong Kong Chinese older adults: family first? Eur J Ageing. 2007;4(4):219–27. 28794791 10.1007/s10433-007-0065-1 PMC5546368

[38] Khami MR, Murtomaa H, Jafarian M, Vehkalahti MM, Virtanen JI. Study motives and career choices of Iranian dental students. Med Princ Pract. 2008;17(3):221–6. 18408391 10.1159/000117796

[39] Watari T, Nagai N, Kono K, Onigata K. Background factors associated with academic motivation for attending medical school immediately after admission in Japan: a single-center study. J Gen Fam Med. 2022;23(3):164–71. 35509336 10.1002/jgf2.528 PMC9062539

[40] Sreeramareddy CT, Shankar PR, Binu VS, Mukhopadhyay C, Ray B, Menezes RG. Psychological morbidity, sources of stress and coping strategies among undergraduate medical students of Nepal. BMC Med Educ. 2007;7:26. 17678553 10.1186/1472-6920-7-26 PMC1951961

[41] Muzafar Y, Khan HH, Ashraf H, Hussain W, Sajid H, Tahir M, Rehman A, Sohail A, Waqas A, Ahmad W. Burnout and its associated factors in medical students of Lahore. Pakistan Cureus. 2015;7(11):e390. 26719833 10.7759/cureus.390 PMC4689594

[42] Almojali AI, Almalki SA, Alothman AS, Masuadi EM, Alaqeel MK. The prevalence and association of stress with sleep quality among medical students. J Epidemiol Glob Health. 2017;7(3):169–74. 28756825 10.1016/j.jegh.2017.04.005 PMC7320447

[43] Mitchell JR, Hart TA, Valcea S, Townsend DM. Becoming the boss: discretion and postsuccession success in family firms. Entrepr Theory Pract. 2009;33(6):1201–18.

[44] Lange T. Job satisfaction and self-employment: autonomy or personality? Small Bus Econ. 2012;38(2):165–77.

[45] Schjoedt L. Entrepreneurial job characteristics: an examination of their effect on entrepreneurial satisfaction. Entrep Theory Pract. 2009;33(3):619–44.

[46] Miller D, Le Breton-Miller I, Lester RH. Family and lone founder ownership and strategic behaviour: social context, identity, and institutional logics. J Manag Stud. 2011;48(1):1–25.

[47] Mahto RV, Khanin D. Satisfaction with past financial performance, risk taking, and future performance expectations in the family business. J Small Bus Manage. 2015;53(3):801–18.

[48] Davis PS, Harveston PD. In the founder’s shadow: conflict in the family firm. Fam Bus Rev. 1999;12(4):311–23.

[49] Cadieux L. Succession in small and medium-sized family businesses: toward a typology of predecessor roles during and after instatement of the successor. Fam Bus Rev. 2007;20(2):95–109.

[50] McBride M. Relative-income effects on subjective well-being in the cross-section. J Econ Behav Organ. 2001;45(3):251–78.

[51] Suls J, Martin R, Wheeler L. Social comparison: why, with whom, and with what effect? Curr Dir Psychol Sci. 2002;11(5):159–63.

[52] Oishi S. The concept of life satisfaction across cultures: an IRT analysis. J Res Pers. 2006;40(4):411–23.

[53] Persson RS. Intellectually gifted individuals’ career choices and work satisfaction: a descriptive study. Gift Talent Int. 2009;24(1):11–23.

[54] Landon BE, Reschovsky J, Blumenthal D. Changes in career satisfaction among primary care and specialist physicians, 1997–2001. JAMA. 2003;289(4):442–9. 12533123 10.1001/jama.289.4.442

[55] Jung JY, Young M. The occupational/career decision-making processes of intellectually gifted adolescents from economically disadvantaged backgrounds: a mixed methods perspective. Gift Child Q. 2019;63(1):36–57.

[56] Goel S, Angeli F, Dhirar N, Singla N, Ruwaard D. What motivates medical students to select medical studies: a systematic literature review. BMC Med Educ. 2018;18(1):16. 29343262 10.1186/s12909-018-1123-4 PMC5772649

[57] Tian L, Pu J, Liu Y, Zhong X, Gui S, Song X, et al. Relationship between burnout and career choice regret among Chinese neurology postgraduates. BMC Med Educ. 2019;19(1):162. 31117998 10.1186/s12909-019-1601-3 PMC6530049

[58] Ritchie A. When practicing medicine runs in the family. Med Econ. 2014;91(22):48–50. 26242067

[59] Gomez-Polo C, Casado AMM, Montero J. Burnout syndrome in dentists: work-related factors. J Dent. 2022;121:104143. 35472454 10.1016/j.jdent.2022.104143

[60] Moro JDS, Soares JP, Massignan C, Oliveira LB, Ribeiro DM, Cardoso M, et al. Burnout syndrome among dentists: a systematic review and meta-analysis. Journal of Evidence-Based Dental Practice. 2022;22(3):101724. 36162888 10.1016/j.jebdp.2022.101724

[61] Long H, Li Q, Zhong X, Yang L, Liu Y, Pu J, et al. The prevalence of professional burnout among dentists: a systematic review and meta-analysis. Psychol Health Med. 2023;28(7):1767–82. 37138501 10.1080/13548506.2023.2208364

[62] Arora S, Knight A. Questionnaire survey of burnout amongst dentists in Singapore. Int Dent J. 2022;72(2):161–8. 34602256 10.1016/j.identj.2021.08.054 PMC9275171

[63] Rotenstein LS, Torre M, Ramos MA, Rosales RC, Guille C, Sen S, et al. Prevalence of burnout among physicians: a systematic review. JAMA. 2018;320(11):1131–50. 30326495 10.1001/jama.2018.12777 PMC6233645

[64] West CP, Dyrbye LN, Shanafelt TD. Physician burnout: contributors, consequences and solutions. J Intern Med. 2018;283(6):516–29. 29505159 10.1111/joim.12752

[65] Cheng FC, Yu-Fong Chang J, Lin TC, Chang WC, Chang YT, Chiang CP. Dentist manpower development and geographical distribution of dentists in Taiwan. J Dent Sci. 2020;15(2):121–31. 32322365 10.1016/j.jds.2020.04.004 PMC7173809

[66] Hui K, Lent RW. The roles of family, culture, and social cognitive variables in the career interests and goals of Asian American college students. J Couns Psychol. 2018;65(1):98–109. 28581305 10.1037/cou0000235

[67] Shen FC, Liao KY, Abraham WT, Weng CY. Parental pressure and support toward Asian americans’ self-efficacy, outcome expectations, and interests in stereotypical occupations: living up to parental expectations and internalized stereotyping as mediators. J Couns Psychol. 2014;61(2):241–52. 24635594 10.1037/a0036219

[68] Fouad NA, Kantamneni N, Smothers MK, Chen Y-L, Fitzpatrick M, Terry S. Asian American career development: a qualitative analysis. J Vocat Behav. 2008;72(1):43–59.

